# Nutritional Metabolites as Biomarkers of Previous Feed Intake in European Rabbit (*Oryctolagus cuniculus*): Applications on Conservation

**DOI:** 10.3390/ani12192608

**Published:** 2022-09-28

**Authors:** Pablo Jesús Marín-García, Lola Llobat, Carlos Rouco, Juan Antonio Aguayo-Adán, Torben Larsen, María Cambra-López, Enrique Blas, Juan José Pascual

**Affiliations:** 1 Department of Animal Production and Health, Veterinary Public Health and Food Science and Technology (PASAPTA), Facultad de Veterinaria, Universidad Cardenal Herrera-CEU, CEU Universities, 46113 Valencia, Spain; 2 Ecology Area, Faculty of Science, University of Cordoba, 14071 Cordoba, Spain; 3 Sociedad, Ecología y Gestión del Medio Ambiente, UCO-IESA, Unidad Asociada al CSIC, 14071 Cordoba, Spain; 4 Department of Animal Science, Aarhus University, DK-8830 Tjele, Denmark; 5 Institute for Animal Science and Technology, Universitat Politècnica de València, Camino de Vera s/n, 46022 Valencia, Spain

**Keywords:** nutrient, energetic, protein, glucose, PUN, NEFA

## Abstract

**Simple Summary:**

This work aims to address the use of biomarkers that can provide us with information on the previous nutrition levels of wild rabbits, a keystone species that has drastically reduced in the last few years. Non-esterified fatty acids (NEFA), plasmatic urea nitrogen (PUN), albumin, glutamate and total protein metabolites were analysed. Additionally, we examined the potential of these metabolites as biomarkers for the nutritional and conservation status of European rabbits to further the biological knowledge of this species and contribute to its conservation.

**Abstract:**

European wild rabbit (*Oryctolagus cuniculus*) populations have drastically reduced, and recently, rabbits have been classed as “endangered” by the IUCN. This animal plays an important ecological role in Mediterranean ecosystems and its introduction could significantly contribute to ecological restoration. Rabbits have high nutrient requirements that apparently cannot be covered in all ecosystems, and there are clues that nutrition can limit their abundance and density. On the other hand, some studies reflect the effects of food availability on the metabolomic status of other animal species, but there are no specific studies on this keystone species. The main aim of this work is to find biomarkers to assess the previous levels of ingestion of European rabbits (*Oryctolagus cuniculus*). To address this gap, gastric content and blood samples were collected from European rabbits (*n* = 99) in a Mediterranean area for the analysis of glucose, non-esterified fatty acids (NEFA), plasmatic urea nitrogen (PUN), albumin, glutamate and total protein metabolites. Depending on their previous feed intake (gastric content and the ratio between the gastric content and the weight of the animal), the animals were divided into two groups (lower and normal previous feed intake). Our work shows that the metabolomic profiles of the animals were affected. Levels of glucose (+82%; *p* = 0.0003), NEFA (−61%; *p* = 0.0040) and PUN (+139%; *p* < 0.001) were different in the animals with lower previous feed intake than the animals with normal previous feed intake. This work summarises that metabolic phenotype can be interesting when seeking to discover the limiting nutrients and food availability in diets that could affect the ecological fitness and conservation of European wild rabbits. It is important to mention that in this work, only the effects on six different metabolites have been analysed and more studies are necessary to complement the knowledge of possible metabolites that indicate the level of ingestion in this species and others. These (and new) biomarkers could be used as a tool to provide information about individual or population characteristics that other physiological parameters cannot detect, improving the conservation physiology field.

## 1. Introduction

European wild rabbits (*Oryctolagus cuniculus*) are defined as a keystone species [1] because they play a relevant ecological role in the Mediterranean ecosystem [2,3]. Some of the reasons for this includes the use of their burrows as a refuge for many species [4,5,6], the effects on the dispersion of Mediterranean plants [2,7], and its role as prey of many carnivores [8], comprising a very important proportion of the diets of the Spanish imperial eagle (*Aquila adalberti*), and the diet of the most threatened feline in the world, the Iberian lynx (*Lynx pardinus*) [9]. Due to different causes [10,11,12], this species has recently been classified as “endangered” by the international Union for Conservation of Nature (IUCN) [13], and the importance of creating specific conservation programs for this species is growing. The loss of a keystone species is critical to an ecosystem’s structure and functioning, and their introduction constitutes a critical point in the ecological restoration of degraded ecosystems [14]. For this reason, the conservation of the rabbit can have a positive impact on the conservation of the Mediterranean ecosystem, a seriously threatened ecosystem [15].

Despite their high number of nutritional adaptations, rabbits have high requirements that apparently cannot be covered in all ecosystems [16,17]. It has been shown that nutrition can limit the abundance and density of this species [18] and an increase in food availability [19] has been shown to be a good management technique [11] and optimal for restocking in some areas, where the estimated mean survival rate for the first 10 days after release was very low (<3%) [20]. In this context, animal nutrition plays a fundamental and limiting role in the recovery of these species [18,21].

Ecological nutrition can use molecular analytical tools to address the management of threatened wildlife [22,23]. In this context, knowing the changes in metabolic phenotype and its relationship with food availability can help increase the understanding of how nutrient availability affects the populations of European rabbits [24]. Different human actions could have indirect consequences on the oxidative status of animals through their effects on the availability of food resources, affecting their biomarkers [25]. In fact, many studies report the effects of food availability on the metabolomic status of animals, for example in *Pygoscelis adeliae* [26], *Anas platyrhynchos* [27], or even *Acrocephalys sechellensis* [28]. Therefore, our hypothesis was that some nutritional metabolites could be affected by previous feed intake, and they could serve as potential biomarkers to determine the previous feeding level in European rabbits.

The main aim of this work was to find biomarkers by analysing metabolites to assess the previous ingestion levels of European rabbit (*Oryctolagus cuniculus*). The potential use of these metabolic biomarkers could improve the emergent field of conservation physiology and ecological nutrition. 

## 2. Materials and Methods

### 2.1. Animal Ethics Statement

The authors confirm that the ethical policies of the journal, as noted on the journal’s author guidelines page, have been adhered to. No ethical approval was required, as no animals were killed specifically for this study. Samples were collected from wild rabbits legally hunted during the official hunting season, in full compliance with the Spanish regulations. No ethical approval by an Institutional Animal Care and Use Committee was deemed necessary.

### 2.2. Animals and Sampling

**Figure 1:**
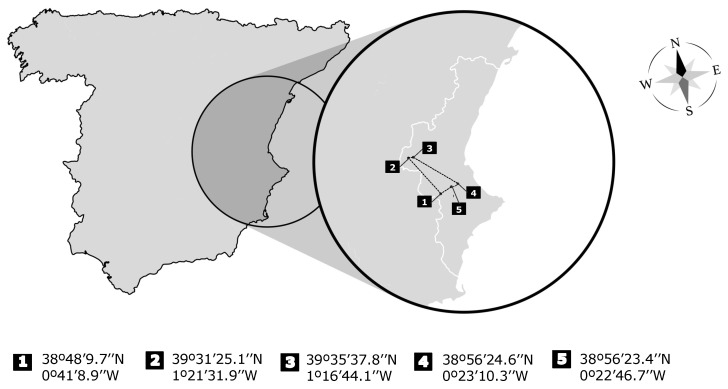
Location of preserves in Spain (and extended Valencian community) and geographic coordinates where European rabbit animals were sampled.

A total of 99 European rabbits were used in this experiment. All animals were obtained as a product of hunting, from five different preserves located in the Valencian community (eastern Spain) (Figure 1). Animals were sampled for digestive content (gastric) and blood. Samples were obtained during May 2021. All samples were obtained during morning hours, at the same time of day (approximately 08:00 a.m.). For each one, the sex (male/female), age (young/adult), reproductive stage (males: non-breeding and in heat; females: non-breeding, pregnant and lactating), state of perirenal thickening, weight, length, the day when the sample was taken, and its location were recorded.

Then, the digestive content of each animal was extracted and weighed to calculate the gastric content weight (the stomach was weighed in its entirety, i.e., the weight of the stomach and its contents). Blood samples were taken from thoracic cavity (1 mL in EDTA vials) and immediately centrifuged for 5 min at 700 G, and the supernatant plasma was extracted. Plasma and gastric content were stored frozen (−20 °C) until further analysis.

### 2.3. Biochemical Analysis of Blood Nutritional Metabolites

Blood plasma glucose, albumin and total protein were determined according to standard procedures (Siemens Diagnostics^®^ Clinical Methods for ADVIA 1800. Foullum, Denmark). 

Non-esterified fatty acids (NEFA) were determined using the Wako, NEFA C ACS-ACOD assay method. Analyses were performed using an ADVIA 1800 ^®^Chemistry System autoanalyser (Siemens Medical Solutions, Tarrytown, NY, USA). 

Glutamate was determined according to the method of Larsen and Fernandéz (2017) [29].

Plasmatic urea nitrogen (PUN) determination was performed using a commercial kit (Urea/BUN-Color, BioSystems S.A., Barcelona, Spain). The samples were defrosted and tempered, after which 1 μL was pipetted into test tubes (each batch included a standard and a blank). Later, 1 mL of reagent A (sodium salicylate 62 mmol/L, sodium nitroprusside 3.4 mmol/L, phosphate buffer 20 mmol/L and urease 500 U/mL) was added to each sample, mixed thoroughly, and incubated for 5 min at 37 °C. Subsequently, 1 mL of reactant B (sodium hypochlorite 7 mmol/L and sodium hydroxide 150 mmol/L) was added, mixed thoroughly, and incubated for another 5 min at 37 °C. Finally, the absorbance of each sample was read at 600 nm against the blank.

Glucose and NEFA were considered as energetic metabolites, while PUN, albumin, total protein and glutamate were considered as protein metabolites.

### 2.4. Statistical Analysis

Preliminary statistical analyses were performed, dividing the population into two groups (low prior intake and normal prior intake). To achieve this goal, gastric content and the ratio between the gastric content and the weight of the animal (more correlated parameter) were analysed. Animals considered to have lower prior feed intake (*n* = 10) had lower gastric content (−42%; *p* = 0.052), and a lower ratio between the gastric content and the weight of the animal (−35%; *p* = 0.0131), compared to those considered to have normal previous feed intake (*n* = 89).

After assigning the experimental groups, gastric content, weight data and nutritional metabolite values were fitted to a normal distribution. After that, an analysis was performed, where NEFA, PUN, albumin, glutamate and total protein were analysed as dependent variables using a GLM model from the Statistical Analysis System [30], in which different groups of previous intake were used as the fixed effect. 

## 3. Results

Table 1 shows the main values (average, range, and coefficient of variation) of the nutritional metabolites monitored in this work, together with the results of the animal’s gastric content measurement. These data showed high variability, revealing the evident differences within the sampled population, including animals being exposed to different previous feed intake, different ages, sexes and several others. Table 1 shows the coefficient of variation for metabolites ranging from 15 to 70% and around 60% for the gastric content measurement. Regarding location, animals with lower previous feed intake were from Preserve 1 (38°48′9.7″ N; 0°41′8.9″ W). This indicates a clear effect of the location on previous ingestion. From this moment on, the differences observed refer to the effect of previous ingestion (lower and normal). Among the rest of the preserves, no significant differences were observed for any observed parameter.

**Figure 2:**
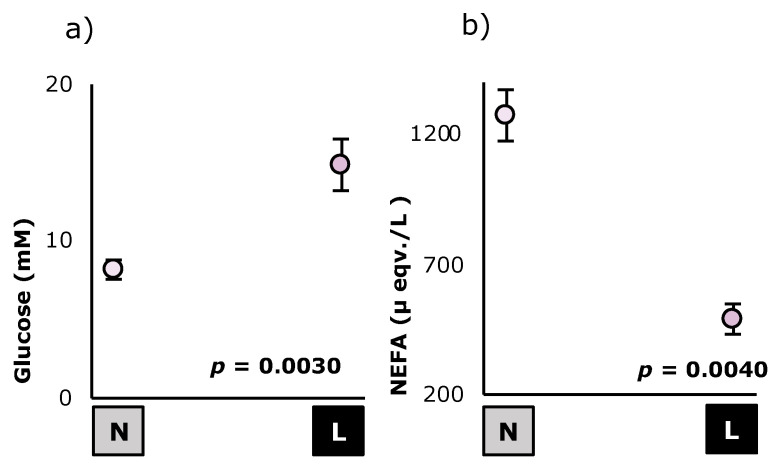
Effect of previous feed intake levels (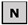 = normal; 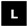 = lower) on the energetic metabolites: Glucose (**a**) and NEFA (**b**) of *Oryctolagus cuniculus* (*n* = 99). Least square means ± standard errors. NEFA: Non-esterified fatty acids.

**Figure 3:**
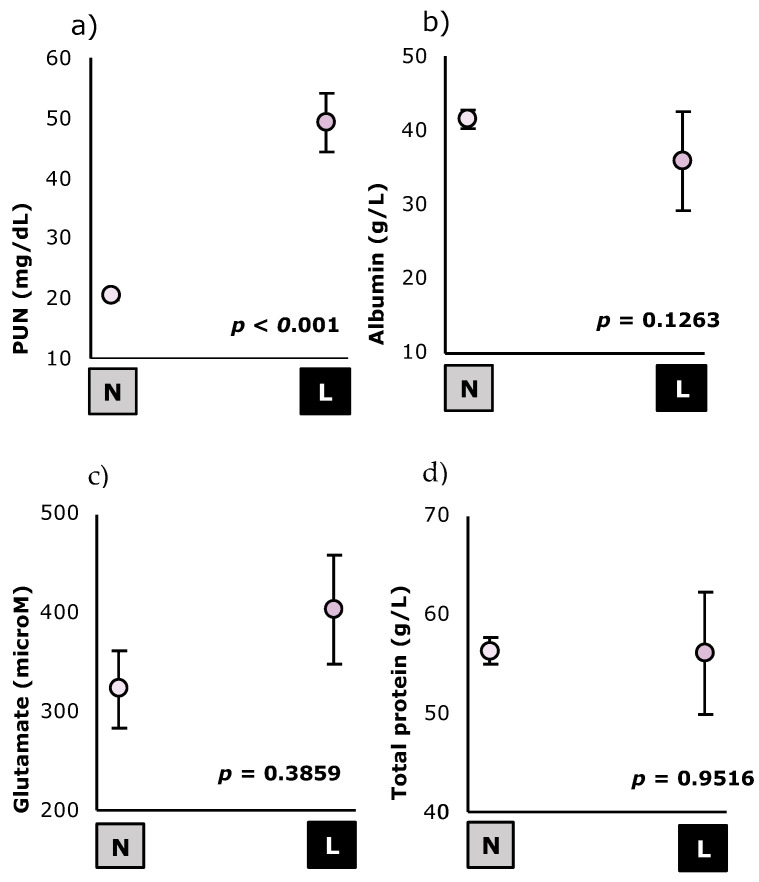
Effect of previous feed intake levels (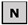 = normal; 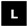 = lower) on the protein metabolites (Plasmatic urea nitrogen (**a**), Albumin (**b**), Glutamate (**c**) and Total protein (**d**)) of *Oryctolagus cuniculus* (*n* = 99). Least square means ± standard errors. PUN: Plasmatic urea nitrogen.

The effect of previous feed intake on the nutritional metabolites is shown in Figure 2 and Figure 3. Because of the previous feed intake, animals in the lower previous feed intake group showed less gastric content (−42%; *p* = 0.005), compared to the animals from the non-restricted group, which potentially had normal feeding behavior. As it can be shown, animals with lower previous feed intake showed higher glucose (+82%; *p* = 0.003) and PUN levels (+138%; *p* < 0.001), and lower NEFA levels (−61%; *p* = 0.0040). 

## 4. Discussion

The main aim of this work was to analyse the effect of previous feed intake on glucose, non-esterified fatty acids (NEFA), plasmatic urea nitrogen (PUN), albumin, glutamate and total protein metabolites. To achieve this goal, it was necessary to use a heterogeneous European rabbit population. This premise was achieved, obtaining very high global ranges and coefficients of variation of all the variables studied. This high variability could indicate the sensitivity of these metabolites, depending on the variables studied. Some metabolites analysed in this work have already been studied before in wild rabbits (albumin and total protein), showing similar values, while PUN levels were lower than those previously observed [31]. However, other parameters have seldom been studied (gastric content, glucose, NEFA and glutamate). 

Gastric content was collected as a measure of previous feed intake, indicating gastric filling and feed availability in each preserve. The literature shows that the weight of gastric content reflects the diurnal rhythm of intake, conditioned by the nutritional behaviour of this species [32,33], considering that gastric content is higher during the morning (when the samples were obtained) than during the night and given that the feeding habits of wild rabbits are even more nocturnal than those of domesticated rabbits. The passage of feed through the stomach of the rabbits is relatively slow, and can reflect the content ingested for 3 and 6 hours prior to the moment of capture, at which time the maximum intake is observed [34], Furthermore, the existing correlation between gastric content and the weight of individuals is similar to that observed with feed ingestion under controlled conditions, so it could be an estimator of previous feed intake. [35] In this context, we can assume that gastric content is related to previous feed intake. Taking into account the animals subjected to normal feeding behaviour, in general, the wild animals showed less gastric content (on av. −50%) than that collected in domestic animals fed *ad libitum* in farm conditions (data compared with those of animals of the same weight) [36]. This may be due to genetic selection, or to the type of food, consisting mainly of plants that have a lower density, which would cause lower weights at the same filling level. As mentioned before, the animals with a low previous intake belonged to a specific reserve; from this moment on, the effects are focused on the animals with different levels of previous intake. On the other hand, the absence of significant differences between the rest of the locations could be due to the different groups of animals, and physiological states, within each of the reserves, in addition to their ability to choose the diets that best suit their nutritional requirements, even if there are differences in the locations sampled.

After a literature search ([37,38,39], as example) and previous results from this same research group ([40,41,42], as example), the six metabolites under study were selected. The inclusion criteria were the evidence of alterations in these metabolites in different conditions (controlled animals and free-range animals) produced by diets with different nutritional levels, which could be related to a better coverage of nutritional requirements in this and other species (rabbits and other species). The effects on other species of the studied metabolites were studied and from there, the following metabolites were selected as the object of study: glucose, NEFA, PUN, albumin, glutamate, and total protein. Next, the main differences in the metabolites, caused by previous ingestion, were recorded. Previous feed intake affected gastric content weight and energetic (glucose and NEFA) and protein blood metabolites (PUN). Animals with low previous feed intake presented markedly lower gastric content. This fact may reinforce the proposal that gastric content is related to previous feed intake. However, the higher glucose levels observed in restricted animals does not agree with previously published data in domestic animals, where feed restriction did not affect glucose levels, [37] or even reduce them [38]. Variables related to feed intake, such as the type of restriction (total or partial), as well as its duration, can affect energetic metabolism. In addition, these studies were carried out in domestic animals, which could explain these differences. Lower levels of NEFA were observed in the animals that were subjected to feed restriction compared to the animals that presented normal feeding behaviour. The reduction in NEFA levels in the animals that were subjected to some type of dietary restriction has already been observed in previous farm situations. This is the case in the work of Ebeid et al. (2012) [38] who observed that partial feed restriction reduced NEFA levels (on av. −21% than animals fed ad libitum). In the case of Harten and Cardoso (2010), [39] NEFA was reduced in domestic animals (−29%; *p* < 0.05) and in wild animals (−42%), when they were subjected to feed restriction. This greater sensitivity in wild animals compared with domestic animals could explain the marked decrease observed in our work (−61%; *p* = 0.004). On the other hand, regarding protein metabolism, PUN was highly affected by feed restriction. A large number of studies have shown that low PUN levels, which correspond to the amount of nitrogen in the form of urea circulating in the bloodstream, could be positively related to performance in domestic animals [40,41,42]. Marín-García et al. (2021) [43] observed that rabbits that were subjected to dietary restriction showed higher (on av. +10%; *p* < 0.05 of all tested diets) PUN levels that animals fed under *ad libitum* conditions [44]. Similar trends (*p* = 0.051) were observed in other works [45]. In general terms, this metabolic alteration could be explained as a catabolism caused by the absence of ingestion, prior to the time of blood extraction. These results suggest a clear effect of both energy and protein catabolism in the animals that demonstrated lower previous feed intake and can, thus, be classified and proposed for use as biomarkers of nutritional status. These data agree with the conclusions observed by Brecchia et al. (2006) [37] who concluded that the nutritional status of rabbits was modified by fasting (although the metabolic response was different in this work).

This work supports the theory that nutrition plays an important role in the metabolomic pathways of animals, and the possibility of using ecological nutrition to unravel the extensive web of nutritional links that drives animals in their interactions with their ecological and social environments [46,47,48], which, in this case, is related with previous feed intake, and its application in conservation physiology.

## 5. Conclusions

The main conclusions drawn from our work are that nutritional metabolites could be used as biomarkers to understand the previous feed intake of European rabbits (Oryctolagus cuniculus). Glucose, NEFA and PUN of the animals with lower levels of previous feed intake were higher, lower and higher, respectively, than the animals with normal previous feed intake. More work is needed to relate the availability of food, its quality, and the adaptive success of this species. The use of these metabolic biomarkers could improve the emergent field of conservation physiology. New biomarkers could complement more conventional physiological parameters, providing information about individual or population characteristics that other physiological parameters cannot detect [25]. In addition, it can provide "clues" about limiting nutrients in populations and determine which plant species should be reintroduced to improve the survival of this endangered species. Finally, it is important to mention that in this work, only a few metabolites have been analysed. Further works are necessary to complement these conclusions, and to allow us to find more biomarkers that can indicate the level of previous feed intake in this and other species, and their relationship with the fields of ecological nutrition and conservation physiology.

## Figures and Tables

**Table 1:** Metabolites values (least square means ± standard errors) of the experimental population. Plasma urea nitrogen (PUN; mg/dL), non-esterified fatty acids (NEFA; µ eqv./L), glucose (mM), albumin (g/L), total protein (g/L) and glutamate (microM) obtained in blood samples of European rabbits (*Oryctolagus cuniculus*).

Metabolites	Range	Values	Coefficient of Variation (%)
PUN	12.95–73.00	24.1 ± 1.32	49.3
NEFA	105–3600	1172 ± 91.8	69.6
Glucose	2.16–30.5	9.07 ± 0.648	62.3
Albumin	25.2–57.1	40.9 ± 1.17	17.1
Total protein	40.9–71.3	56.3 ± 8.36	14.8
Glutamate	74–589	329 ± 22.1	37.5
Gastric content weight (g)	2.17–109.8	22.39 ± 2.50	59.5

## Data Availability

The datasets of the current study are available from the corresponding author upon reasonable request.
